# Blimp-1 impairs T cell function via upregulation of TIGIT and PD-1 in patients with acute myeloid leukemia

**DOI:** 10.1186/s13045-017-0486-z

**Published:** 2017-06-19

**Authors:** Liuluan Zhu, Yaxian Kong, Jianhong Zhang, David F. Claxton, W. Christopher Ehmann, Witold B. Rybka, Neil D. Palmisiano, Ming Wang, Bei Jia, Michael Bayerl, Todd D. Schell, Raymond J. Hohl, Hui Zeng, Hong Zheng

**Affiliations:** 1 Institute of Infectious Diseases, Beijing Ditan Hospital, Beijing Key Laboratory of Emerging Infectious Diseases, Capital Medical University, Beijing, China; 2 Penn State Cancer Institute, Penn State University College of Medicine, Hershey, PA USA; 3 Depatment of Medical Oncology, Thomas Jefferson University, Philadelphia, PA USA; 4 Department of Public Health Sciences, Penn State University College of Medicine, Hershey, PA USA; 5 Department of Pathology, Penn State Hershey Medical Center, Penn State University College of Medicine, Hershey, PA 17033 United States; 6 Department of Microbiology and Immunology, Penn State University College of Medicine, Hershey, PA USA

**Keywords:** Blimp-1, TIGIT, PD-1, T cell exhaustion, Acute myeloid leukemia (AML)

## Abstract

**Background:**

T cell immunoglobulin and immunoreceptor tyrosine-based inhibitory motif (ITIM) domain (TIGIT) and programmed cell death protein 1 (PD-1) are important inhibitory receptors that associate with T cell exhaustion in acute myeloid leukemia (AML). In this study, we aimed to determine the underlying transcriptional mechanisms regulating these inhibitory pathways. Specifically, we investigated the role of transcription factor B lymphocyte-induced maturation protein 1 (Blimp-1) in T cell response and transcriptional regulation of TIGIT and PD-1 in AML.

**Methods:**

Peripheral blood samples collected from patients with AML were used in this study. Blimp-1 expression was examined by flow cytometry. The correlation of Blimp-1 expression to clinical characteristics of AML patients was analyzed. Phenotypic and functional studies of Blimp-1-expressing T cells were performed using flow cytometry-based assays. Luciferase reporter assays and ChIP assays were applied to assess direct binding and transcription activity of Blimp-1. Using siRNA to silence Blimp-1, we further elucidated the regulatory role of Blimp-1 in the TIGIT and PD-1 expression and T cell immune response.

**Results:**

Blimp-1 expression is elevated in T cells from AML patients. Consistent with exhaustion, Blimp-1^+^ T cells upregulate multiple inhibitory receptors including PD-1 and TIGIT. In addition, they are functionally impaired manifested by low cytokine production and decreased cytotoxicity capacity. Importantly, the functional defect is reversed by inhibition of Blimp-1 via siRNA knockdown. Furthermore, Blimp-1 binds to the promoters of PD-1 and TIGIT and positively regulates their expression.

**Conclusions:**

Our study demonstrates an important inhibitory effect of Blimp-1 on T cell response in AML; thus, targeting Blimp-1 and its regulated molecules to improve the immune response may provide effective leukemia therapeutics.

**Electronic supplementary material:**

The online version of this article (doi:10.1186/s13045-017-0486-z) contains supplementary material, which is available to authorized users.

## Background

Acute myeloid leukemia (AML) is the most common acute leukemia in adults with around 20,000 new diagnoses each year in the USA. The mainstream of treatment for AML is induction chemotherapy followed by post-remission consolidation. Allogeneic hematopoietic stem cell transplantation in many clinical settings can significantly improve survival. However, the overall prognosis remains poor with a 5-year survival rate of only 25%. Novel effective leukemia therapeutics is urgently needed.

Modulating the immune response to improve anti-tumor immunity provides a promising strategy for cancer treatment [1]. Studies using reagents inhibiting negative immune regulatory pathways, such as programmed cell death protein 1 (PD-1), have achieved great success [2–5]. This strategy targets T cell exhaustion, a status of T cell dysfunction that contributes to compromised anti-tumor T cell responses. Exhausted T cells gradually lose their capacity for cytokine production and cell killing, eventually undergo apoptosis and deletion [6]. Upregulation of PD-1 and other inhibitory pathways such as T cell immunoglobulin domain and mucin domain 3 (TIM-3), 2B4, Lymphocyte-activation gene 3 (LAG-3), and T cell immunoglobulin and immunoreceptor tyrosine-based inhibitory motif (ITIM) domain (TIGIT) is not only a hallmark, but also an important mechanism involved in the development of T cell exhaustion [7–18]. Studying the role of inhibitory pathways in AML is appealing. Several reports including ours have shown that inhibitory receptors including PD-1, TIM-3, and TIGIT are elevated on T cells and associate with immune suppression in AML [19–26]. Combined blockade of PD-1 and TIM-3 pathways synergistically reduced tumor burden and leukemia death in a mouse model of AML [21]. These data suggest that targeting the inhibitory pathways to restore T cell function and anti-tumor immune response may represent an effective leukemia therapy. Moving this strategy forward to clinical applications is under active development, but it is also important to understand the transcriptional mechanisms involved in the regulation of these inhibitory receptors in AML, which is currently unknown.

B lymphocyte-induced maturation protein 1 (Blimp-1) is a zinc finger-containing transcription factor functioning as a decision maker for memory B cell differentiation [27, 28]. Recent studies in mouse models of infection uncovered its crucial role in regulating CD8^+^ T cell exhaustion [29–31]. Here, we examine the effect of Blimp-1 in the pathogenesis of AML using blood samples collected from a cohort (*n* = 24) of patients with AML at initial diagnosis. We demonstrate an inhibitory role for Blimp-1 on T cell response in AML. Elevated expression of Blimp-1 in T cells associates with upregulation of inhibitory receptors and reduced T cell capacity of cytokine production and cytotoxicity, features which are consistent with exhaustion. Importantly, Blimp-1 knockdown by siRNA reverses these functional defects. In addition, Blimp-1 binds to the promoters of PD-1 and TIGIT and upregulates their expression, suggesting that the suppressive effect of Blimp-1 on the T cell response is mediated by its transcriptional regulation of PD-1 and TIGIT. Our studies demonstrate that Blimp-1 is an important regulator of T cell exhaustion in AML and thus an attractive target for effective leukemia therapeutics.

## Methods

### Patients

Peripheral blood collected from AML patients were obtained from the tissue bank maintained by the Penn State Hershey Cancer Institute of Penn State University College of Medicine, Hershey, PA. The study was approved by the Institutional Review Board of Penn State University College of Medicine. Full informed consent was obtained from all patients. Samples from 24 patients (10 males and 14 females, age 57 ± 15 years, range, 23–77 years) diagnosed with AML per WHO classification were used in the study. Samples of 25 healthy volunteers (13 males and 12 females, age 55 ± 15 years, range, 21–77 years) were obtained as controls.

### Immunofluorescence staining and flow cytometric analysis

For surface staining, cells were incubated at room temperature with human Fc block (BD Biosciences, San Diego, CA, USA) and followed by staining with directly conjugated mAbs for 30 min at 4 °C. The mAbs used were anti-human CD3-BV605, CD4-V500, CD8-APC-H7, CD45RA-BV421, CCR7-PerCp-Cy5.5, PD-1-PE-Cy7, CD160-Alexa Fluor 488 (BD Biosciences), CD4-FITC, TIM-3-BV421, 2B4-PerCp-Cy5.5 (BioLegend, San Diego, CA, USA), and TIGIT-APC (eBioscience, San Diego, CA, USA). Data acquisition was performed on a LSR Fortessa flow cytometer (BD Biosciences). FlowJo Software (Tree Star, Ashland, OR, USA) was used in data analysis.

### SmartFlares

Lyophilized SmartFlare probe (Merck Millipore, Guyancourt, France) was used to detect Blimp-1 mRNA following the manufacturer’s instruction.

### In vitro stimulation and intracellular staining

PBMCs were stimulated with anti-CD3/CD28 (2 and 5 μg/mL), plus Golgiplug (BD Pharmingen) for 5 h before intracellular staining Blimp-1-PE, IFN-γ-PE-CF594, and IL-2-PerCp-Cy5.5 (BD Pharmingen). For perforin study, perforin-PE-CF594 (BD Pharmingen) was used. A Fixable Viability Dye eFluor 450 (eBioscience) was used to assess cell viability.

### siRNA transfection

SMARTpool of siRNA for Blimp-1 and control were obtained from GE Dharmacon RNA Technologies (GE Dharmacon, Lafayette, CO, USA). Control and specific siRNAs were added at a final concentration of 1 μM per well for 72 h. For functional assays, cells were further stimulated with anti-CD3/CD28 for 5 h, followed by flow analysis.

### Plasmid construction, transfection, and real-time PCR

*PRDM1α* plasmid (RGS-6xHis-BLIMP-1-pcDNA3.1-) was a gift from Adam Antebi [32]. *PRDM1β* cDNA was cloned into pcDNA3.1+ plasmid. The *PD-1* gene promoter (−1063/+70 bp relative to the transcription start site) and *TIGIT* promoter (−2228/+76 bp) were cloned into pGL3-basic. *PRDM1α* and *PRDM1β* plasmids were transfected using Lipofectamine 3000 (Thermo Fisher Scientific, Waltham, MA, USA). Specific transcripts were quantified by real-time PCR with TaqMan probes according to the manufacturer’s instructions (Thermo Fisher Scientific).

### Luciferase reporter assay

293T cells were transfected with a mixture of the indicated expression plasmids. After 24 h, luciferase assays were performed using a dual-Luciferase Reporter Assay System (Promega, Madison, WI, USA) according to the manufacturer’s instructions.

### Chromatin immunoprecipitation (ChIP) assay

ChIP assays were conducted as previously described [33]. Briefly, T cells were stimulated in vitro with anti-CD3 [34] for 48 h followed by cross-linking, sonication, and chromatin immunoprecipitation with antibodies to Blimp-1 or normal goat IgG (Abcam, Cambridge, UK). DNA was then quantified by real-time PCR. Primer sequences were provided in Additional file 1: Supplemental data.

### Statistical analysis

GraphPad5 (GraphPad Software, La Jolla,CA, USA) was used for statistical calculations. The normality of each continuous variable was evaluated using the Kolmogorov–Smirnov test. For data distributed normally, the comparison of variables was performed using unpaired or paired (where specified) Student’s *t* test. For data not distributed normally, the comparison of variables was performed with a Mann–Whitney *U* test or a Wilcoxon signed-rank test for unpaired and paired data, respectively. Comparisons of categorical patient characteristics were analyzed using Fisher’s exact test. To evaluate correlation, Pearson’s correlation coefficients were used. All tests are two-tailed with *P* values less than 0.05 considered statistically significant.

## Results

### Blimp-1 is upregulated in T cells from AML patients

**Fig. 1:**
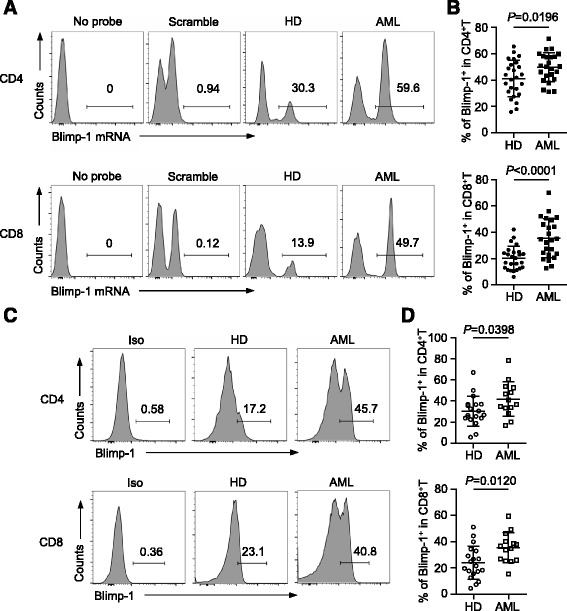
Blimp-1 is elevated in T cells from AML patients. PBMCs collected from AML patients at initial diagnosis as well as healthy donors were assessed. **a**, **b** Expression of Blimp-1 mRNA was assessed by SmartFlare probe followed by flow cytometry analysis. **a** Representative histograms displaying the expression of Blimp-1 mRNA gated on CD4^+^ (*left*) and CD8^+^ T cells (*right*). Data from one healthy donor and one AML patient are shown. Scrambled SmartFlare probes were set as negative controls. **b** Plot of percentages of Blimp-1^+^ cells in CD4^+^ and CD8^+^ T cells from healthy donors (*n* = 25) vs. AML patients (*n* = 24). **c**, **d** Expression of Blimp-1 protein was assessed by intracellular staining. **c** Representative flow cytometry data. **d** Plot of percentages of Blimp-1^+^ cells in CD4^+^ and CD8^+^ T cells from healthy donors (*n* = 19) vs. AML patients (*n* = 14). Each *spot* represents an individual patient or healthy donor. *P* values were obtained by unpaired *t* test.

To determine the effect of Blimp-1 on the T cell response in patients with AML, we first assessed the expression of Blimp-1 mRNA in both CD4^+^ and CD8^+^ T cells. PBMCs collected from 24 AML patients at initial diagnosis were examined. Samples from 25 age- and gender-matched healthy donors (HD) served as controls. We used a novel technology, the SmartFlare system [35], to detect Blimp-1 mRNA by flow cytometry. Importantly, this nanoparticle-based system allows us to test the transcripts within individual living cells. We observed a significant elevation of Blimp-1 mRNA in both CD4^+^ and CD8^+^ T cells from AML patients, compared with those from HD. The mean frequency (±SD) of Blimp-1^+^ cells among CD4^+^ T cells was 41.2 ± 14.8% vs. 49.8 ± 9.5%, *P* = 0.0196 (Fig. 1a, b), while those of CD8^+^ T cells was 20.3 ± 7.9% vs. 35.4 ± 17.3%, *P* < 0.0001 (Fig. 1a, b). To determine the expression of Blimp-1 in protein level, we performed intracellular staining of Blimp-1 in both CD4^+^ and CD8^+^ T cells. Consistent with the mRNA data, the frequency of Blimp-1^+^ T cells was significantly higher in AML patients, compared with those in HD (Fig. 1c, d). This data indicates a potential role for Blimp-1 in AML.

### Elevated Blimp-1 expression on CD4^+^ T cells correlates with high circulating blasts in AML patients

We next analyzed the correlation of Blimp-1 expression with the clinical characteristics in AML patients. Based on the level of Blimp-1 mRNA expression on T cells, we defined high-Blimp-1 (Blimp-1 ≥49.8% of CD4^+^ T cells, ≥35.4% of CD8^+^ T cells) vs. low-Blimp-1 (Blimp-1 <49.8% of CD4^+^ T cells, <35.4% of CD8^+^ T cells) subgroups in AML patients. The median values of Blimp-1 expression on T cells of the 24 AML patients evaluated in our study were used to establish the cutoff values. Patients with high Blimp-1 expression in CD8^+^ T cells had comparable clinical characteristics with that of low Blimp-1 expression (Additional file 1: Table S1). We further dissected the association of Blimp-1 expression in CD4^+^ T cells to clinical characteristics (Table 1). We found no significant difference between the two groups (high Blimp-1 vs. low Blimp-1) in terms of age, gender, and cytogenetic features. However, we observed that patients expressing a high frequency of Blimp-1 mRNA in CD4^+^ T cells presented with significantly higher white blood counts (WBC) at initial diagnosis. A high, yet comparable proportion of WBCs were leukemia blasts in both the high- and low-Blimp-1 groups. Therefore, the absolute blast counts in the peripheral blood were significantly higher in patients with high Blimp-1-expressing CD4^+^ T cells compared to that of patients with low Blimp-1 expression (Table 1).

**Table 1:** High Blimp-1 expression on CD4^+^ T cells associates with increased blast in AML

Total	High-Blimp-1	Low-Blimp-1	*P* value
(*n* = 24)	(*n* = 13)	(*n* = 11)	
Age, years			
Median	61	59	0.797
Range	23–75	24–77	
Gender			
Male	6	4	0.628
Female	7	7	
WBC, × 10^9^/l			
Median	82.9	24.9	0.033
Range	5.8–364.6	5.1–87	
PB blast, %			
Median	68	73	0.721
Range	1.9–93.8	18–90.8	
Absolute blast counts, × 10^9^/l			
Median	41	16	0.026
Range	0.1–342	3–47	
BM blast, %			
Median	60	64.5	0.626
Range	1.5–88	34.5–79	
Cytogenetics^a^			
Adverse	6	6	0.799
Intermediate	5	4	
Favorable	1	1	

*WBC* white blood cell, *ANC* absolute neutrophil counts, *PB* peripheral blood, *BM* bone marrow, *ITD* internal tandem duplication.

^a^Risk stratification is based on the 2017 European Leukemia Net Recommendations. Clinical information for risk stratification was not available for one patient, thus data of 23 (12 of high Blimp-1, 11 of low Blimp-1) are shown

### Expression of Blimp-1 correlates with the upregulation of inhibitory receptors on T cells from AML patients

**Fig. 2:**
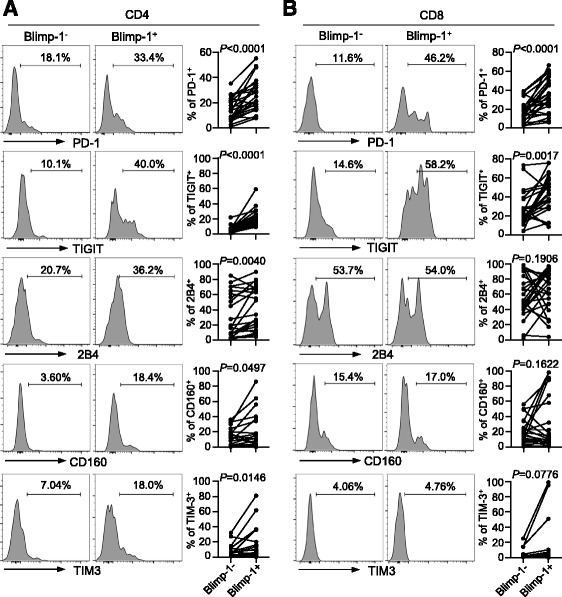
Expression of Blimp-1 correlates with the upregulation of inhibitory receptors on T cells from AML patients. Flow cytometry analysis of expression of PD-1, TIGIT, 2B4, CD160, and TIM-3 on Blimp-1^−^ vs. Blimp-1^+^ T cells from AML patients (*n* = 24). Blimp-1 mRNA expression are detected by SmartFlare. Data of CD4^+^ (**a)** and CD8^+^ (**b)** T cells are shown. Panels on the *right* of each set of representative histograms are plots of expression of each receptor on Blimp-1^−^ vs. Blimp-1^+^ T cells. *P* values were obtained by paired *t* test and Wilcoxon signed-rank test.

To examine whether there is an association between the expression of Blimp-1 and multiple inhibitory receptors in AML, PBMCs collected from AML patients were co-cultured with Blimp-1 probe followed by co-staining with antibodies to inhibitory receptors including PD-1, TIGIT, 2B4, CD160, and TIM-3. Multi-channel flow cytometry analysis was performed to detect the expression of inhibitory receptors on T cells that co-express Blimp-1 mRNA. As shown in Fig. 2a, Blimp-1^+^ CD4^+^ T cells expressed significantly higher levels of all inhibitory receptors tested than Blimp-1^−^ CD4^+^ T cells. CD8^+^ T cells demonstrated a similar expression pattern although only TIGIT and PD-1 achieved statistical significance (Fig. 2b). Expression of Blimp-1 was positively correlated with the expression of PD-1 and TIGIT in both CD4^+^ and CD8^+^ T cells from AML (Additional file 1: Figure S1). Thus, expression of Blimp-1 associates with the upregulation of multiple inhibitory receptors on T cells from AML patients, suggesting a potential effect of Blimp-1 in suppressing T cell function in AML.

### Expression of Blimp-1 in CD8^+^ T cells associates with increased differentiation of terminally differentiated effector T cells in AML

**Fig. 3:**
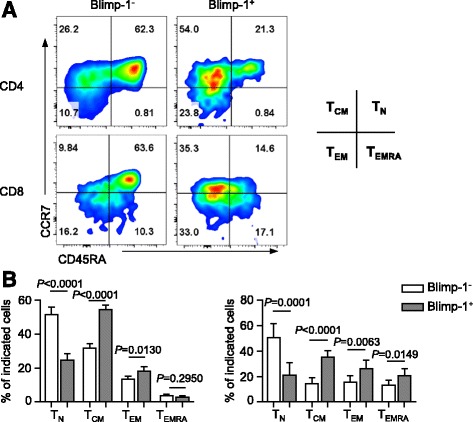
Expression of Blimp-1 in CD8^+^ T cells associates with increased differentiation of terminal differentiated effector T cells in AML. Distribution of T_N_, T_CM_, T_EM_, and T_EMRA_ within Blimp-1^−^ vs. Blimp-1^+^ T cells from AML patients (*n* = 14) are assessed by flow cytometry. Both CD4^+^ and CD8^+^ T cells are evaluated. Representative flow data (**a**) and plots (**b**) of percentage of each T cell differentiation subset among Blimp-1^−^ vs. Blimp-1^+^ T cells are shown. *P* values were obtained by paired *t* test and Wilcoxon signed-rank test.

We further performed phenotypic analyses to evaluate the differentiation status of T cells that express Blimp-1 mRNA in patients with AML. Based on the expression of CD45RA and CCR7, T cells can be divided into four subpopulations: naïve T cells (T_N_, CCR7^+^CD45RA^+^), central memory T cells (T_CM_, CCR7^+^CD45RA^−^), effector memory T cells (T_EM_, CCR7^−^CD45RA^−^), and terminally differentiated effector cells (T_EMRA_, CCR7^−^CD45RA^+^) [36–39]. Consistent with a previous report that Blimp-1 is mainly expressed in T cells post antigen stimulation [29, 40], we observed that the majority of Blimp-1^+^ T cells are antigen-experienced cells in AML patients. Blimp-1^+^ cells among both CD4^+^ and CD8^+^ T cells showed significantly increased T_CM_ and T_EM_ compared to that of Blimp-1^−^ cells. In contrast, Blimp-1^−^ T cells were mostly naive (Fig. 3). Importantly, the frequency of T_EMRA_ was significantly higher in Blimp-1^+^ CD8^+^ T cells (Fig. 3). T_EMRA_ are considered as terminal effector cells with limited functional capacity; thus, these data provide further support that Blimp-1 negatively influences T cell response in AML.

### Blimp-1^+^ T cells from AML patients display functional defects manifested by reduced cytokine production and cytotoxic capacity

**Fig. 4:**
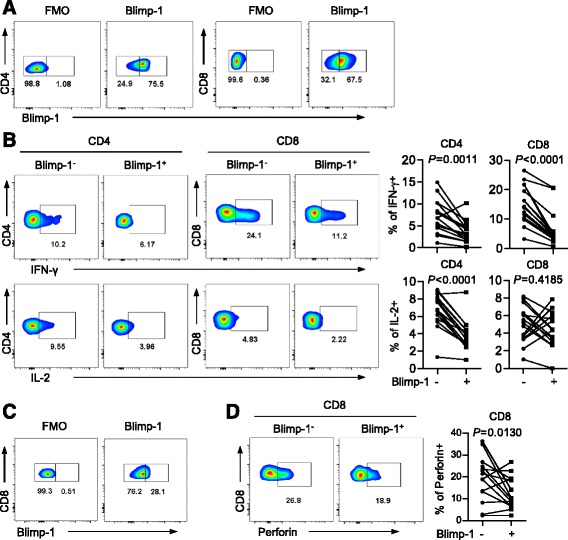
Blimp-1^+^ T cells from AML patients display functional defects by showing less cytokine production and capacity of cytotoxicity. **a**, **b** PBMCs collected from AML patients were stimulated in vitro with anti-CD3 and anti-CD28 before intracellular staining with Blimp-1, IFN-γ, and IL-2. **a** Flow cytometry data showing Blimp-1 expression in both CD4^+^ and CD8^+^ T cells. Fluorescence-minus-one (FMO) stains were used as negative controls. **b** Intracellular production of IFN-γ and IL-2 among Blimp-1^−^ vs. Blimp-1^+^ T cells from AML patients (*n* = 15) were dissected. Shown are representative dot plots (*left*) and a plot of frequency (*right*) for IFN-γ and IL-2, respectively. Data of both CD4^+^ and CD8^+^ T cells are shown. **c** Flow cytometry data showing Blimp-1 expression in CD8^+^ T cells without in vitro stimulation. **d** Intracellular stain of perforin by Blimp-1^+^CD8^+^ vs. Blimp-1^−^CD8^+^ T cells from AML patients (*n* = 15). Representative flow data (*left*) and plot of the percentage of perforin^+^ CD8^+^ T cells (*right*) are shown. *P* values were obtained by paired *t* test.

To evaluate the functional status of Blimp-1^+^ T cells from the AML patients, we tested cytokine release upon in vitro stimulation with anti-CD3 and anti-CD28. Intracellular production of IFN-γ and IL-2 were assessed by flow cytometry analysis gated on Blimp-1^+^ vs. Blimp-1^−^ T cells. Of note, we were not able to examine the mRNA expression of Blimp-1 in this experiment as the RNA probe leaks post-permeabilization during intracellular staining for cytokines. Therefore, Blimp-1^+^ T cells were defined by intracellular staining of Blimp-1 protein (Fig. 4a). As shown in Fig. 4b, the Blimp-1^+^ subpopulation of both CD4^+^ and CD8^+^ T cells had significantly lower intracellular IFN-γ compared to Blimp-1^−^ T cells. Blimp-1^+^ CD4^+^ T cells also displayed less IL-2 production compared to that of Blimp-1^−^ CD4^+^ T cells. We next assessed the cytotoxic potential by examining the level of perforin expression in each T cell subpopulation. Without in vitro stimulation, CD8^+^ T cells appear to express less Blimp-1 (Fig. 4c). Importantly, we observed a significant lower expression of perforin and Granzyme B in Blimp-1^+^ CD8^+^ T cells, compared to that in Blimp-1^−^ CD8^+^ T cells (Fig. 4d, Additional file 1: Figure S2A). Collectively, these results demonstrate that Blimp-1^+^ T cells have a decreased function compared to Blimp-1^−^ T cells in AML patients.

### Inhibition of Blimp-1 improves T cell function

**Fig. 5:**
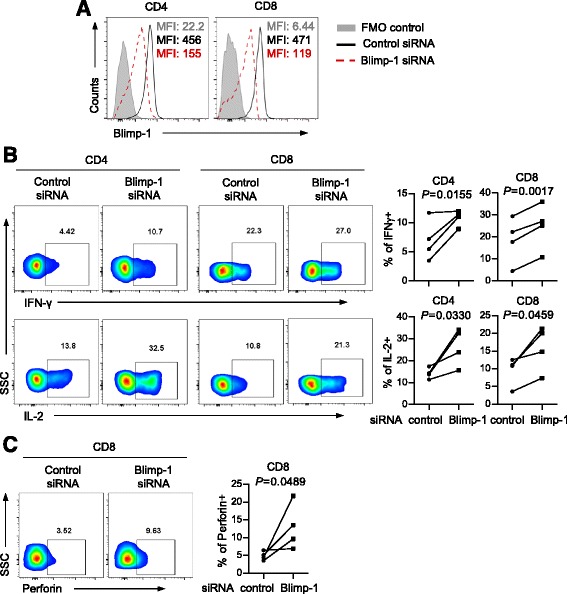
Blimp-1 knockdown with siRNA increases cytokine production and cytotoxicity capacity in T cells from AML patients. **a** Histograms of Blimp-1 MFI show the efficiency of Blimp-1 siRNA knockdown. **b** Intracellular cytokine production by purified CD4^+^ and CD8^+^ T cells from AML patients (*n* = 4) upon anti-CD3/anti-CD28 stimulation. **c** Intracellular production of perforin by purified CD8^+^ T cells from AML patients (*n* = 4) upon Blimp-1 knockdown. Shown are representative flow data (*left*) and plot of frequency (*right*). *P* values were obtained by paired *t* test.

To further dissect the regulatory effect of Blimp-1 on T cell function, we used a specific siRNA to knockdown Blimp-1 expression in T cells from the AML patients. The expression of Blimp-1 was reduced by 60% in both CD4^+^ and CD8^+^ T cells after transfection with Blimp-1 siRNAs (Fig. 5a). Intracellular cytokine staining assays were performed on T cells upon in vitro stimulation with anti-CD3 and anti-CD28. We observed a significant increase of IFN-γ and IL-2 production after Blimp-1 inhibition by siRNA; this occurred in both CD4^+^ and CD8^+^ T cells (Fig. 5b). Consistently, CD8^+^ T cells expressed increased levels of perforin and Granzyme B upon Blimp-1 knockdown, indicating an improved cytotoxic capacity (Fig. 5c, Additional file 1: Figure S2B). Of note, Blimp-1 did not appear to regulate apoptosis as we observed no change of CD95 expression on T cells upon Blimp-1 knockdown (Additional file 1: Figure S3). These important data demonstrate a pivotal role for Blimp-1 in inhibiting cytokine release and cytotoxic capacity, thus suppressing T cell function in AML patients.

### Blimp-1 is a direct transcriptional regulator of PD-1 and TIGIT

**Fig. 6:**
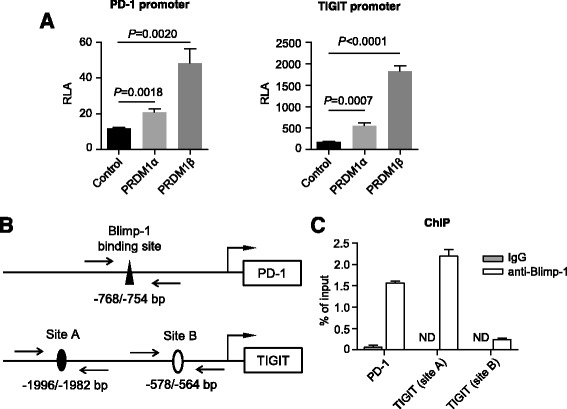
Blimp-1 directly binds to the promoter of PD-1 and TIGIT genes. **a** 293 T cells were transfected with *PD-1* promoter (−1063/+76 bp) or *TIGIT* promoter (−2228/+70 bp), *PRDM1α* or *PRDM1β* expressing plasmid, and pRL-TK for 24 h. Luciferase activities were measured and normalized to that of Renilla luciferase. **b** Schematic diagram of the PCR amplicons for the putative Blimp-1 binding sites in *PD-1* and *TIGIT* promoters. **c** ChIP assays were performed using T cells purified from PBMCs of a healthy donor. T cells were stimulated with anti-CD3 antibody for 48 h. Putative Blimp-1 binding sites in the promoters of *PD-1* and *TIGIT* were examined by qPCR using specific primers as described in the section of methods. Nonspecific goat IgG was used as a negative control.

Our study demonstrated a tight correlation of Blimp-1 expression to that of PD-1 and TIGIT in AML (Fig. 2), we hypothesize that Blimp-1 suppresses T cell function by regulating the expression of these two inhibitory receptors. There are two isoforms of Blimp-1 detected in the T cells from AML: isoforms 1 and 2, which were encoded by transcript variant positive regulatory domain containing 1 alpha (*PRDM1α*) and *PRDM1β*, respectively (Additional file 1: Figure S4). Blimp-1 isoform 2 has a truncated PR domain, lacking the N-terminal 101 amino acids of isoform 1. We performed a luciferase assay using PD-1 and TIGIT luciferase reporters. Both *PRDM1α* and *PRDM1β* exert a significant transcriptional activity for the expression of PD-1 and TIGIT. *PRDM1β* appears to be more dominant (Fig. 6a). To test whether there is a direct binding of Blimp-1 to the promoter of *PD-1* and *TIGIT*, we analyzed the promoter sequences of *PD-1* and *TIGIT*. One and two binding sites for Blimp-1 were located on the promoter of PD-1 and TIGIT, respectively (Fig. 6b). In a ChIP assay using T cells purified from PBMCs of a healthy donor, we observed a clear interaction between Blimp-1 and its binding site on the *PD-1* promoter. Between the two putative binding sites on the *TIGIT* promoter, Blimp-1 binds to site A, but not site B (Fig. 6c). Thus, there is a direct binding of Blimp-1 to the promoters of *PD-1* and *TIGIT*.

**Fig. 7:**
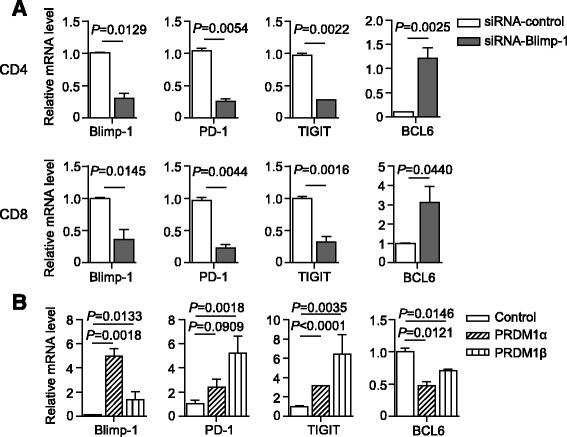
Blimp-1 positively regulates the expression of PD-1 and TIGIT. **a** Purified CD4^+^ and CD8^+^ T cells from AML patients (*n* = 3) were transfected with indicated siRNA. Expression of mRNA for Blimp-1, PD-1, TIGIT, and BCL-6 upon Blimp-1 knockdown was assessed by real-time PCR. **b** MT4 cells were transfected with *PRDM1α* and *PRDM1β* plasmids for 48 h. The mRNA levels of Blimp-1, PD-1, TIGIT, and BCL6 were quantified by real-time PCR. Values were normalized to those of GAPDH and expressed relative to negative control. *P* values were obtained by unpaired *t* test.

To determine whether Blimp-1 regulates the expression of PD-1 and TIGIT in AML, we first assessed the effect of Blimp-1 knockdown on the mRNA expression of PD-1 and TIGIT using T cells purified from PBMCs of AML patients. Consistent with our findings in Fig. 5a, Blimp-1 knockdown efficiently silenced its mRNA (Fig. 7a). Importantly, we observed a significant decrease of PD-1 and TIGIT mRNA in both CD4^+^ and CD8^+^ T cells upon Blimp-1 knockdown. As a control, BCL-6 mRNA was increased upon Blimp-1 knockdown (Fig. 7a). We next performed a study overexpressing the transcripts *PRDM1α* and *PRDM1β* in a human T cell line MT4 cell. We observed a significant elevation of both PD-1 and TIGIT mRNA. In contrast, BCL-6 mRNA was decreased when *PRDM1α* or *PRDM1β* was overexpressed (Fig. 7b).

Taken together, these data demonstrate that the transcription factor Blimp-1 can bind to the promoters of *PD-1* and *TIGIT* and positively regulate the expression of these two inhibitory receptors.

## Discussion

Blimp-1, encoded by the *PRDM1* gene, was initially identified as a transcriptional repressor regulating terminal differentiation of B cells into plasma cells [41]. The effect of Blimp-1 in lymphoproliferative disorders has been well studied [42–47]. Recent studies using mouse models of virus infection elucidated its role in T cell differentiation. During acute viral infections, Blimp-1 promotes the differentiation of CD8^+^ T cells into short-lived terminal effectors while dampening the formation of long-lived central memory T cells [40, 48–50]. During chronic viral infection, Blimp-1 enhances expression of inhibitory receptors and promotes development of T cell exhaustion [29, 51, 52]. Notably, haploinsufficient mice which had intermediate expression of Blimp-1 controlled chronic virus infection better than either wild type or Blimp-1fully deficient mice, indicating that a moderate amount of Blimp-1 facilitates effector mechanisms without causing T cell exhaustion [29, 30]. These findings suggest a complex role for Blimp-1 in regulating T cell response. Although well studied in viral infection, the T cell regulatory role of Blimp-1 in tumor immunity has not been fully defined and the effect of Blimp-1 on anti-leukemia response is unknown. In this study, phenotypic and functional analyses of PBMCs collected from AML patients were performed. We focused on dissecting the role of Blimp-1 in modulating the T cell response in AML. Our study demonstrates that expression of Blimp-1 in both CD4^+^ and CD8^+^ T cells is significantly increased in AML patients compared to that in healthy donors. Consistent with exhaustion, Blimp-1^+^ T cells express high levels of co-inhibitory receptors such as PD-1 and TIGIT. In addition, they are phenotypically skewed toward terminal effector differentiation and functionally impaired in their production of cytokines and potential for cytotoxicity. Importantly, the functional defect is reversed by inhibition of Blimp-1 via siRNA knockdown. To our knowledge, this study is the first to display an immune suppressive role of Blimp-1 in AML. Our finding suggests that Blimp-1 associates with T cell exhaustion and suppresses T cell function, which may subsequently impair anti-leukemia immune response. Therefore, targeting Blimp-1 may provide effective therapeutics for AML.

We observed a wide variation of Blimp-1 expression in T cells among AML patients. Clinically, the initial presentation of AML is highly heterogeneous [53]. Some patients seek medical attention earlier during the disease course due to their high sensitivity to leukemia-related symptoms or occasionally incidental abnormal laboratory findings; others present later when the leukemia has developed for a longer period of time. The large variation of Blimp-1 expression among the AML patients may represent their different disease status. In fact, we found a significant association of Blimp-1 expression with the number of circulating leukemia blast. Patients who express high levels of Blimp-1 in their CD4^+^ T cells present with high blast counts, indicating a correlation of Blimp-1 expression to late phase leukemia development. This situation might provide persistent leukemia antigen that is ideal for induction of T cell exhaustion, which is consistent with our finding that Blimp-1^+^ T cells associate with exhaustion and display functional impairment. Thus, we speculate that treatment approaches targeting T cell exhaustion may be more effective in patients with higher expression of Blimp-1 as T cells in this patient population are more likely exhausted. Therefore, testing Blimp-1 expression in T cells might provide a crucial biomarker for effective leukemia treatment. Although promising, further studies of large size of samples are needed to make a definitive conclusion.

The mechanisms by which Blimp-1 regulates T cell responses are not fully understood. In our study, we observed a strong correlation between Blimp-1 expression and upregulation of inhibitory receptors such as PD-1 and TIGIT. Several studies have demonstrated an important role of PD-1 in inhibiting anti-leukemia T cell responses [20, 21, 24]. In addition, our recent study revealed that TIGIT contributes to T cell impairment in AML and associates with poor clinical outcomes [26]. We hypothesize that in AML, Blimp-1 suppresses T cell function through positive regulation of these inhibitory pathways. In the present study, we demonstrated a strong binding of Blimp-1 protein to the promoters of the genes encoding PD-1 and TIGIT. Importantly, inhibition of Blimp-1 by siRNA knockdown significantly decreased mRNA expression of PD-1 and TIGIT in T cells collected from AML patients. Consistently, cells overexpressing Blimp-1 showed upregulation of PD-1 and TIGIT. Therefore, Blimp-1 is a transcriptional regulator for these two important inhibitory receptors. This likely contributes to the mechanisms by which Blimp-1 suppresses T cell function in AML. An equally important question is how and why Blimp-1 is upregulated in AML. In viral infection, Blimp-1 expression is induced during T cell activation upon viral antigen stimulation [31]. Cytokines including IL-2 have been reported to be crucial mediators for the upregulation of Blimp-1. In AML, it has been observed that serum level of IL-2 is increased in AML patients, and the level is particularly higher in patients with high WBC at initial presentation [54]. Consistently, we observed a positive correlation between the high level of WBC and Blimp-1 expression in our study. We speculate that IL-2 and/or other cytokines may contribute to the regulation of Blimp-1 in AML. Further studies are warranted to address this important question.

In contrast to our finding that Blimp-1 upregulates the expression of PD-1, Lu et al. have reported that Blimp-1 inhibits CD8^+^ T cell expression of PD-1[55]. Of note, their conclusion was drawn from a study of acute viral infection, in which PD-1 was increased shortly (hours) after antigen stimulation. The regulation mechanisms may be significantly different in the setting of chronic infections or cancer. Consistent with our finding, it has been reported that PD-1^+^ T cells expressed a high level of Blimp-1 in patients with chronic lymphocytic leukemia [56]. In addition, studies using mouse models of viral infection have demonstrated that Blimp-1 enhanced the expression of inhibitory receptors on exhausted T cells during chronic viral infection and conditional deletion of Blimp-1 reversed this process [29]. Collectively, these observations highlight the importance of the context (disease status)-specific transcriptional mechanisms during T cell differentiation.

Majority of studies demonstrate a dominant role of CD8^+^ T cells in host defense. Features of CD8^+^ T cell exhaustion and its effect on dysfunctional immune status have been extensively investigated [57]. Recent observations of CD4^+^ T cell exhaustion in chronic viral infections suggest that CD4^+^ T cells are also crucial for optimal infection control [58, 59]. Most recently, Hwang et al. reported that Blimp-1 is upregulated and acts as a critical regulator for CD4^+^ T cell exhaustion during chronic toxoplasmosis. Conditional deletion of Blimp-1 in CD4^+^ T cells regained CD8^+^ T cell function and improved infection control [60]. Contributions of CD4^+^ T cell in leukemia are not well defined. Our findings demonstrate that, in addition to causing CD8^+^ T cell dysfunction, Blimp-1 plays an equally important role in mediating CD4^+^ T cell suppression in AML. Blimp-1 upregulates co-inhibitory receptors and associates with functional defect in both CD4^+^ and CD8^+^ T cells. Interestingly, high Blimp-1 expression in CD4^+^, not CD8^+^ T cells, correlates with high circulating leukemia blast (Table 1), suggesting a potential unique contribution of CD4^+^ T cell dysfunction in AML pathogenesis.

## Conclusions

Taken together, we demonstrate an inhibitory effect of Blimp-1 on T cell response in AML patients. Blimp-1 can do so by transcriptionally upregulating inhibitory receptors including PD-1 and TIGIT. A clinical correlative study showed an association between the elevated Blimp-1 expression and high circulating blasts in AML patients. Our findings have significant clinical impact as Blimp-1 may be a useful biomarker and an important target for effective novel leukemic therapeutics.

## Additional file

Additional file 1: Figure S1. Expression of Blimp-1 positively correlates with TIGIT and PD-1 expression in AML. Figure S2: Blimp-1+ T cells express less Granzyme B, which is partially reversed by Blimp-1 knockdown. **Figure S3.** The expression of CD95 was not affected by Blimp-1 knockdown. **Table S1.** Blimp-1 is expressed as two transcript variants PRDM1α and PRDM1β in T cells from AML patients (PDF 451 kb)

## Abbreviations

AML: Acute myeloid leukemia
Blimp-1: B lymphocyte–induced maturation protein 1
LAG-3: Lymphocyte-activation gene 3
PD-1: Programmed cell death protein 1
PRDM1α: Positive regulatory domain containing 1 alpha
TIGIT: T cell immunoglobulin and ITIM domain
TIM-3: T cell immunoglobulin domain and mucin domain 3
WBC: White blood counts
