# Supplementary material for: Blimp-1 impairs T cell function via upregulation of TIGIT and PD-1 in patients with acute myeloid leukemia
Source: J Hematol Oncol. 2017 Jun 19;10:124. doi: 10.1186/s13045-017-0486-z (PMC5477125; doi:10.1186/s13045-017-0486-z)
Supplement: Additional file 1: Figure S1. — Expression of Blimp-1 positively correlates with TIGIT and PD-1 expression in AML. Figure S2: Blimp-1+ T cells express less Granzyme B, which is partially reversed by Blimp-1 knockdown. Figure S3. The expression of CD95 was not affected by Blimp-1 knockdown. Table S1. Blimp-1 is expressed as two transcript variants PRDM1α and PRDM1β in T cells from AML patients (PDF 451 kb) [file 13045_2017_486_MOESM1_ESM.pdf]

Supplemental Materianls and Methods

SmartFlares

Lyophilized SmartFlare probe (Merck Millipore, Guyancourt, France) was reconstituted with nuclease-free water to a stock solution of 100 nM that was stored in the dark at room temperature. The stock solution was diluted with PBS 1:3 to a working solution. PBMCs collected from healthy donors (HD) and AML patients were cultured with RPMI-1640 medium containing 10% FBS and 2mM glutamine at a concentration of 5×10^5 cells/mL. After verifying cell viability, 196 μL of cell suspension was added per well of a 96-well plate to yield 1×10^5 cells/well. 4 μL of SmartFlare working solution was added to each well and the suspension mixed by gentle pipetting. Plates were incubated at 37°C for 16 hours. Cells were then harvested into staining buffer and stained with anti-surface marker antibodies.

In vitro stimulation and intracellular staining

PBMCs were cultured in RPMI-1640 medium (GIBCO) containing 10% FBS with control and specific siRNA for 72 hours, followed by stimulation with anti-CD3/CD28 (2 μg/mL and 5 μg/mL) antibodies, plus Golgiplug (BD Pharmingen) for 5 hours. The cells were then surface stained with CD4-FITC, CD8-APC-H7, CD95-BV421 (BD Pharmingen), and intracellularly stained with Blimp-1-PE. A violet amine reactive dye (Invitrogen) was used to assess cell viability.

RT-PCR

$CD4^+$ and $CD8^+$ T cells purified from fresh PBMCs of AML patients. Total RNA was isolated with RNeasy Micro Kit (Qiagen, Venlo, Netherlands) according to the manufacturer's instructions, and then reverse-transcribed to cDNA using the High Capacity cDNA Reverse Transcription kit (Thermo Fisher Scientific). Transcripts of PRDM1α and PRDM1β were quantified with semi-quantitative RT-PCR. GAPDH was taken as an internal reference. Primer sequences were as follows: for PRDM1α, 5'–GTTCCTAAGAACGCCAACAGG–3' (sense) and 5'–GCAAAGTCCCGACAATACCAC–3' (anti-sense), the amplicon length is 239 bp; for PRDM1β, 5'–GTTAATCGGTTTGAGGCA–3' (sense) and 5'–AAATGTTAGAACGGTAGAGGT–3' (anti-sense), the amplicon length is 501 bp; for GAPDH, 5'–GAAGGTGAAGGTCGGAGTC–3' (sense) and 5'–GAAGATGGTGATGGGATTTC–3' (anti-sense), the amplicon length is 226 bp.

Real-time PCR for ChIP assay

After chromatin immunoprecipitation with antibodies to Blimp-1, DNA was subjected to real-time PCR with SYBR Green PCR Master Mix (Thermo Fisher Scientific). Primers were

designed to amplify the region of the human *PD-1* and *TIGIT* promoters containing putative Blimp-1 binding sites. Primer sequences were as follows: *PD-1*, 5'–GCTAGGAAAGACAATGGTGGCA–3' (sense) and 5'–ACAGAGGGCAGTGGTGGGAC–3' (anti-sense); *TIGIT* site A, 5'–ATCAGATAAGGAGGGCAGAATG–3' (sense) and 5'–TTCATAGGGTTGTGAGGATTTA–3'(anti-sense); site B, 5'–TCCAGATAAACAAGAC AAAAGAAA–3' (sense) and 5'–GAAGAATGTGCAATGATAAGAGAA–3'(anti-sense).

Supplemental Figure Legend

Supplemental Figure 1. Expression of Blimp-1 positively correlates with TIGIT and PD-1 expression in AML.

Expression of Blimp-1 mRNA was assessed by SmartFlare. Co-stain of multiple inhibitory receptors (PD-1, TIGIT, 2B4, CD160, and TIM-3) was performed before flow cytometry study and correlative analysis between Blimp-1 and each inhibitory receptor. Data of both $CD4^+$ and $CD8^+$ T cells are shown.

Supplemental Figure 2. Blimp-1$^+$ T cells express less Granzyme B, which is partially reversed by Blimp-1 knockdown.

(A) Flow cytometry analysis of Granzyme B expression was performed on PBMCs collected from AML patients (n=15) at initial diagnosis. Representative histograms and plot of percentages display the expression of Granzyme B gated on $CD8^+$ T cells. (B) Blimp-1 knockdown with siRNA increases Granzyme B expression. Representative histograms and plot of percentages show the intracellular production of Granzyme B by purified $CD8^+$ T cells from AML patients (n=4) upon Blimp-1 knockdown. Data are presented as Mean ± SD. *P* values were obtained by paired *t* test.

Supplemental Figure 3. The expression of CD95 was not affected by Blimp-1 knockdown.

$CD4^+$ or $CD8^+$ T cells were sorted from PBMCs of AML patients (n=4) and then cultured with control or Blimp-1 siRNA for 72 hours followed by 5 hours of anti-CD3/anti-CD28 stimulation. The expression of CD95 was analyzed by flow cytometry.

Supplemental Figure 4. Blimp-1 is expressed as two transcript variants PRDM1α and PRDM1β in T cells from AML patients.

Expressions of PRDM1α and PRDM1β were detected using RT-PCR. GAPDH was taken as an internal reference. The products were analyzed by 1.5% agarose gel electrophoresis.

Supplemental table 1. No correlation between Blimp-1 expression in $CD8^+$ T cells and clinical characteristics in AML patients.

Total (n=24)	High-Blimp-1 (n=10)	Low-Blimp-1 (n=14)	P-value
**Age, years**			
Median	60	62.5	0.292
Range	23-66	30-77	
**Gender**			
Male	6	4	0.135
Female	4	10	
**WBC, × 10^9/l**			
Median	34.8	43.5	0.519
Range	12.4-223	5.9-364.6	
**PB blast, %**			
Median	73.4	59.5	0.263
Range	37.7-87.8	1.9-98	
**Absolute blast counts, × 10^9/l**			
Median	26	22	0.440
Range	10-152	0.1-342	
**BM blast, %**			
Median	57.3	65.3	0.517
Range	1.5-88	20-82.5	
**Cytogenetics**			
Adverse	4	7	0.804
Intermediate	5	5	
Favorable	1	1	

Abbreviations: WBC, white blood cell; ANC, absolute neutrophil counts; PB, peripheral blood; BM, bone marrow; ITD, internal tandem duplication. * Information for Risk Stratification (ELN 2016) was not available for one patient, thus data of 23 patients (10 of high-Blimp-1, 13 of low-Blimp-1) are shown.

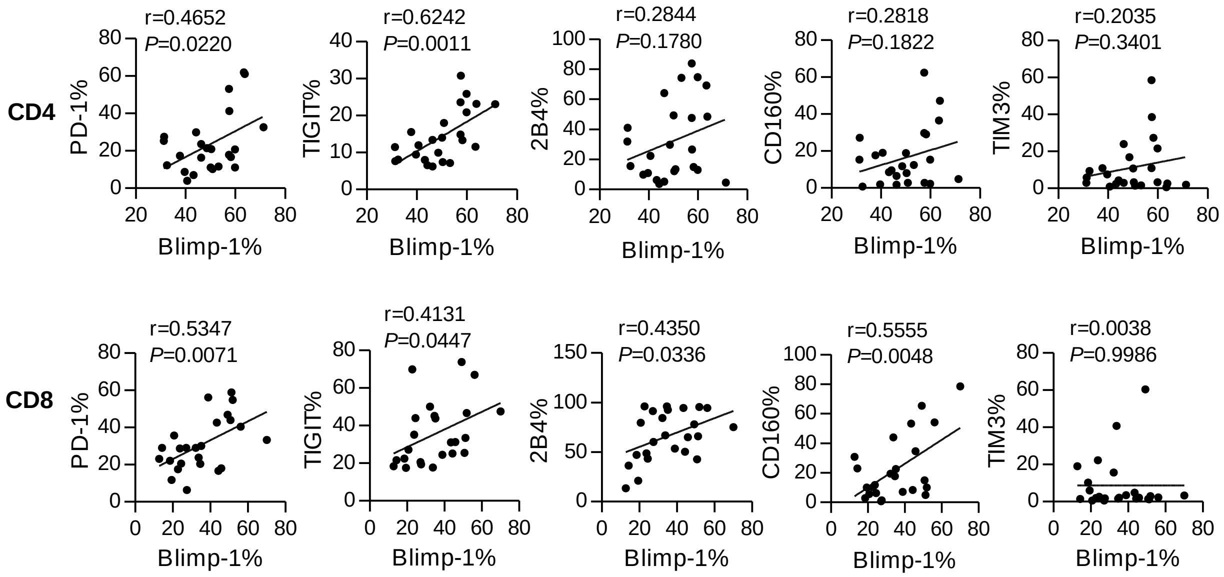

Supplemental figure 1

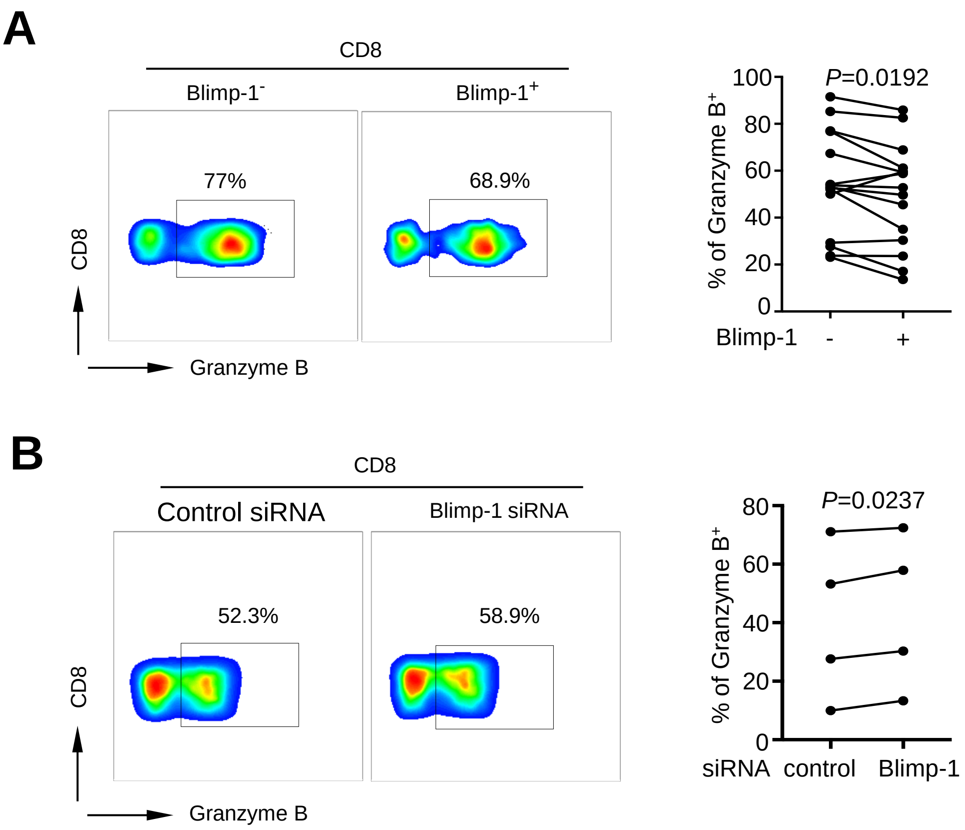

Supplemental Figure 2.

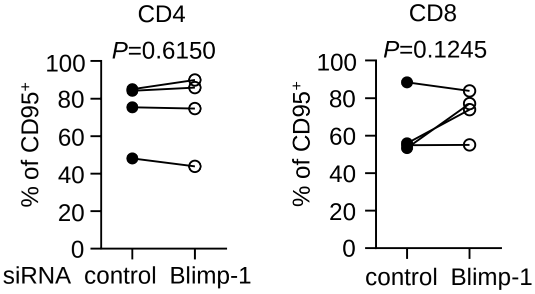

Supplemental figure 3

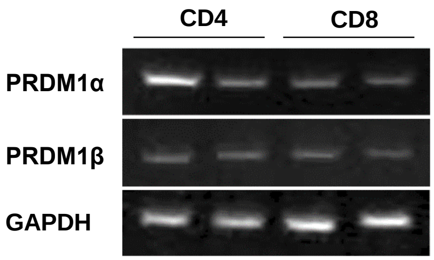

Supplemental figure 4
